# Pyramid Inter-Attention for High Dynamic Range Imaging

**DOI:** 10.3390/s20185102

**Published:** 2020-09-07

**Authors:** Sungil Choi, Jaehoon Cho, Wonil Song, Jihwan Choe, Jisung Yoo, Kwanghoon Sohn

**Affiliations:** 1 Department of Electrical and Electronic Engineering, Yonsei University, Seoul 03722, Korea; csi6570@yonsei.ac.kr (S.C.); rehoon@yonsei.ac.kr (J.C.); swonil92@yonsei.ac.kr (W.S.); 2 Samsung Electronics, Suwon 16677, Korea; jihwan.choe@samsung.com (J.C.); jisung.yoo@samsung.com (J.Y.)

**Keywords:** HDR imaging, attention mechanisms, optical flow

## Abstract

This paper proposes a novel approach to high-dynamic-range (HDR) imaging of dynamic scenes to eliminate ghosting artifacts in HDR images when in the presence of severe misalignment (large object or camera motion) in input low-dynamic-range (LDR) images. Recent non-flow-based methods suffer from ghosting artifacts in the presence of large object motion. Flow-based methods face the same issue since their optical flow algorithms yield huge alignment errors. To eliminate ghosting artifacts, we propose a simple yet effective alignment network for solving the misalignment. The proposed pyramid inter-attention module (PIAM) performs alignment of LDR features by leveraging inter-attention maps. Additionally, to boost the representation of aligned features in the merging process, we propose a dual excitation block (DEB) that recalibrates each feature both spatially and channel-wise. Exhaustive experimental results demonstrate the effectiveness of the proposed PIAM and DEB, achieving state-of-the-art performance in terms of producing ghost-free HDR images.

## 1. Introduction

Humans can see in a wide range of lighting conditions because the human eye adjusts constantly to a broad range of natural luminance values in the environment. However, standard digital cameras typically fail to capture images with sufficient dynamic range because of the limited ranges of sensors. To alleviate this issue, high-dynamic-range (HDR) imaging has been developed to improve the range of color and contrast in captured images [1]. Given a series of low-dynamic-range (LDR) images captured at different exposures, an HDR image is produced by merging these LDR images.

Traditional methods for producing HDR images [2,3] are based on the assumption that the images are globally registered, i.e., there is no camera or object motion between images with different exposure values. However, misalignments are inevitable in the presence of foreground motion and small camera motions, thus they usually suffer from ghosting artifacts. Many solutions [4,5,6,7,8,9,10,11,12,13,14,15] have been developed to overcome this limitation. HDR imaging reconstruction relying on pixel rejection [4,5,6,7,8] simply rejects pixels in misaligned regions as outliers. Other methods rely on registration [9,10,11,12,13,14,15,16] to reconstruct HDR images by searching for the best matching regions in LDR images.

Based on the recent development of convolutional neural networks (CNNs), the performance of HDR imaging using CNNs [17,18,19,20,21,22] has been significantly improved. Eilertsen et al. [22] proposed an autoencoder network to produce HDR images from only a single image. Endo et al. [17] proposed to synthesize LDR images captured with different exposures (i.e., bracketed images) and then reconstruct an HDR image by merging the synthesized images. However, the reliance on a single input LDR image cannot handle the highly contrastive scenes since it is an ill-posed problem. Kalantari et al. [19] attempted to handle the misalignment problem of dynamic scenes by implementing the classical optical flow algorithm [23] as an alignment process. However, the classical optical flow algorithm shows large alignment errors, which products artifacts in misalignment region. In addition, the classical optical flow algorithm requires significant computational time. Although Wu et al. [20] formulated HDR imaging as an image translation problem without alignment, they failed to reconstruct the details of an HDR image in occluded regions. Yan et al. [21] proposed an attention-guided deep network for suppressing misaligned features during the merging process to avoid ghosting artifacts. However, their method [21] still suffers from ghosting artifacts, because they excluded alignment between LDR images in the presence of camera motion or foreground motion.

**Figure 1:**
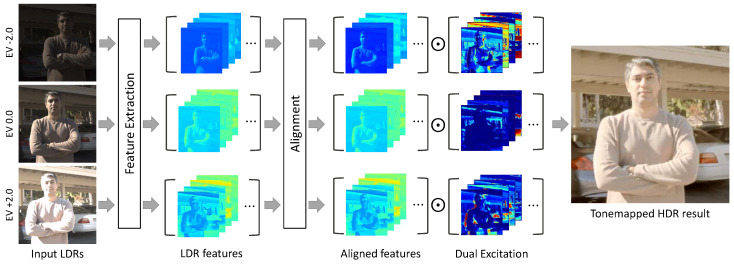
Given low-dynamic-range (LDR) images of a dynamic scene as inputs, the proposed method first generates the features using shared feature extraction network. Before merging them, the alignment network aligns non-reference features to a reference feature (i.e., EV0) using the pyramid inter-attention module (PIAM). In the merging process, we recalibrate these features to concentrate on more useful elements for producing a ghost-free high-dynamic-range (HDR) image, using both spatial and channel excitations. Finally, the proposed method outputs a tonemapped HDR image.

In this paper, we propose a novel end-to-end flow-based HDR method, including pyramid inter-attention module (PIAM) and dual excitation block (DEB) for the alignment and merging processes, respectively. Our method is the first to jointly estimate the correspondence between LDR images and reconstruct HDR images. Specifically, during the alignment process, we can align the non-reference feature to a reference feature by leveraging the PIAM, as shown in Figure 1. Furthermore, we use the DEB to recalibrate the LDR features spatially and channel-wise for boosting the representation of features for generating ghost-free HDR images in the merging process. The main contributions of this paper can be summarized as follows:

- We propose a novel CNN-based framework for ghost-free HDR imaging by leveraging pyramid inter-attention module (PIAM) which effectively aligns LDR images.
- We propose a dual excitation block (DEB), which recalibrates features both spatially and channel-wise by highlighting the informative features and excluding harmful components.
- Extensive experiments on HDR datasets [11,19,24] demonstrate that the synergy between the two aforementioned modules enables our framework to achieve state-of-the-art performance.

## 2. Related Work

### 2.1. HDR Imaging without Alignment

We first review HDR imaging algorithms using the assumption that input LDR images are globally registered. Early work presented by Mann and Picard [2] attempted to combine differently exposed images to obtain a single HDR image. Debevec and Malik [3] recovered camera response function using differently exposed photographs with a static camera. Unger et al. [25] designed an HDR imaging system using a highly programmable camera unit and multi-exposure images. Khan et al. [26] computed the probabilities of pixels for part of an image background by iteratively weighting the contribution of each pixel. Jacobs et al. [5] removed ghosting artifacts by addressing brightness changes. Pece and Kautz [7] proposed a motion map to compute median threshold bitmaps for each image. Heo et al. [8] assigned weights to emphasize well-exposed pixels using Gaussian-weighted distance. Zhang and Cham [4] detected movement using quality measures based on image gradients to generate a weighting map. Lee et al. [27] and Oh et al. [28] explored rank minimization in HDR deghosting to detect motion and reconstruct HDR images. However, these solutions are impractical because they are not able to handle moving objects or camera motion.

### 2.2. HDR Imaging with Alignment

To solve the misalignment of dynamic scenes for HDR imaging, some approaches align LDR images prior to reconstructing an HDR image by applying dense correspondence algorithms (i.e., optical flow). Bogoni [10] aligned LDR images via warping using local motion vectors, which are estimated based on optical flow algorithm. Kang et al. [9] exploited the optical flow algorithm after performing exposure correction between LDR images. Jinno and Okuda [29] estimated dense correspondences based on a Markov random field model. Gallo et al. [14] proposed a fast non-rigid registration method for input images where small motion exists between them. However, these approaches cannot handle ghosting artifacts in the presence of large foreground motion, because they use a simple merging process for combining aligned LDR images.

There have been many attempts to integrate alignment and HDR reconstruction into a joint optimization process. Sen et al. [11] proposed a patch-based energy-minimization method that integrates alignment and reconstruction into a joint optimization process. Hu et al. [15] decomposed the optimization problem by using image alignment based on brightness and gradient consistency. Hafner et al. [12] proposed an energy-minimization approach that simultaneously calculates HDR irradiance and displacement fields. Despite these improvement of HDR imaging, such methods still have limitations when large motions and saturation exist in LDR images.

### 2.3. Deep-Learning-Based Methods

Recently, several deep CNN-based methods for HDR imaging [17,19,20,21,22] have been proposed. First, Eilertsen et al. [22] proposed a method for reconstructing HDR images from single LDR images using an autoencoder network. The method proposed by Endo et al. [17] predicts multiple LDR images with different exposures from a single LDR image, then reconstructs a final HDR image by merging the predicted images using a deep learning network. These methods have a limitation in that they use only a single LDR image, which makes it difficult to synthesize the details of an HDR image.

Kalantari et al. [19] attempted to solve the misalignment of LDR images by using an off-the-shelf optical flow algorithm [23]. They then merged the aligned LDR images to obtain an HDR image using CNNs. However, the optical flow algorithm [23] has a large computational time. Wu et al. [20] proposed a non-flow-based translation network that can elucidate plausible details from LDR inputs and generate ghost-free HDR images. Yan et al. [21] proposed an attention network to suppress the undesirable features due to the misalignment or saturation to avoid the ghosting artifacts. Although the methods discussed above represent remarkable advances in HDR imaging, they [20,21] cannot fully exploit the information from all LDR images. In contrast to these recent works [19,20,21], we incorporate a simple yet effective alignment network into the HDR imaging network to reconstruct details of HDR images by aligning LDR features.

### 2.4. Optical Flow

Alignment between LDR images is a key factor for generating ghost-free HDR images. The optical flow algorithm can be to perform alignment by finding the correspondence between the images. As a classical optical flow algorithm, the SIFT-flow algorithm [23] is an optimization-based algorithm for finding the optical flow between images. However, optimization-based methods require large computational times. Inspired by the success of CNNs, FlowNet [30] was the first end-to-end learning approach for optical flow. This method estimates the dense optical flow between two images based on a U-Net autoencoder architecture [31]. FlowNet 2.0 [32] stacks several basic FlowNet models for iterative refinement and significantly improves accuracy. Recently, PWC-Net [33] was proposed to warp features in each feature pyramid in a coarse-to-fine approach and achieve state-of-the-art performance with a lightweight framework. However, these deep-learning-based flow estimation methods for estimating optical flows cannot handle the large object motions.

### 2.5. Attention Mechanisms

Attention mechanisms have provided significant performance improvements for many computer vision tasks, such as image classification [34], semantic segmentation [35], and image generation [36,37]. In the works by Zhang et al. [36] and Wang et al. [34], self-attention mechanisms were proposed for modeling long-range dependencies solve the problem of limited local receptive fields that many deep generative model have. For stereoscopic super-resolution tasks, Wang et al. [38] proposed a parallax-attention module for finding stereo correspondence. They found reliable correspondences with smaller computational cost than other stereo matching networks [39,40,41] by leveraging a parallax-attention mechanism. Inspired by attention mechanisms, we effectively find correspondence between the LDR images captured in dynamic scenarios for reconstructing HDR images. Then we align the LDR features using the correspondences for fully exploiting these features. Although our method and Yan et al. [21] use the term "attention", there is a significant difference between these methods. The attention network proposed by the Yan et al. [21] focuses on highlighting meaning features for HDR imaging. In contrast, our method aligns LDR images for fully exploiting them for HDR imaging via inter-attention maps.

## 3. Proposed Method

### 3.1. Overview

**Figure 2:**
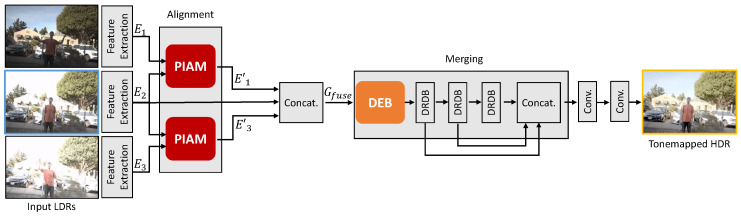
Overall framework for the proposed method. Our framework consists of three sections: a feature extraction network, alignment network, and merging network. First, we extract features from multiple LDR images using a feature extraction network. The alignment network, termed as pyramid inter-attention module (PIAM), is used to align the features from the feature extraction network. In the merging network, the dual excitation block (DEB) recalibrates features both spatially and channel-wise. A dilated residual dense block (DRDB) is used to learn hierarchical features for HDR imaging effectively.

An overview of the proposed method is presented in Figure 2. Using a set of LDR images $\left\{I_{1},I_{2},\ldots ,I_{k}\right\}$ of a dynamic scene sorted by their exposure values, the proposed method aims to reconstruct a ghost-free HDR image $H_{r}$ that is aligned to the reference LDR image $I_{r}$. First, we apply gamma correction [19,20,21] for mapping each LDR image $I_{i}$ into the HDR domain according to its exposure time $t_{i}$ (i.e., $J_{i}={I_{i}}^{\gamma }/t_{i}$, where we set γ to 2.2 in this work), as a preprocessing step. Similar to previous approaches [19,20,21], the input for the proposed method is a concatenation of $I_{i}$ and $J_{i}$, where i=1,2,3. After preprocessing, we feed each input into the feature extraction network, which is composed of several combinations of convolution and rectified linear unit (ReLU) function, resulting in $E_{i}$.

To exploit the features $E_{o}$, o∈{1,3} from other LDR images (i.e., non-reference images), the alignment network warps other features $\left\{E_{1},E_{3}\right\}$ by leveraging the proposed a pyramid inter-attention module (PIAM). The reference-aligned features and the reference feature are then merged to synthesize the details of the target HDR image. Although the alignment network aligns these features, alignment errors still exist in case of homogeneous regions or repetitive patterns. To handle this problem, we propose a dual excitation block (DEB) to recalibrate features for highlighting the informative features and excluding harmful features. Finally, the dilated residual dense blocks (DRDB) are used to learn hierarchical features for HDR imaging effectively.

### 3.2. Alignment Network

Since the features from LDR images are not aligned, we conduct alignment for fully exploiting them prior to merging features. When camera motion or a moving object exists in a scene, the alignment process is a key factor for reconstructing an HDR image. Unlike the method proposed by [19], which uses the classical optical flow algorithm [23], we propose a novel alignment network, called PIAM. Before we describe the details of the PIAM, we will illustrate inter-attention module (IAM).

#### 3.2.1. Inter Attention Module

**Figure 3:**
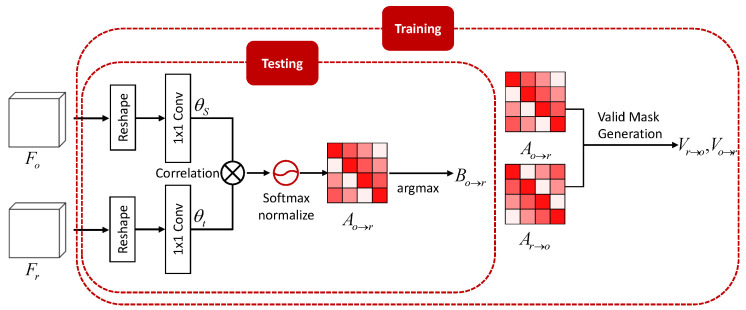
Illustration of the inter-attention module (IAM). Using discriminative features $\left\{F_{r},F_{o}\right\}$, it outputs a hard inter-attention map $B_{o\to r}$ for alignment in testing phase. Additionally, soft inter-attention maps $\left\{A_{o\to r},A_{r\to o}\right\}$ and valid masks $\left\{V_{o\to r},V_{r\to o}\right\}$ are used for photometric loss for training the IAM.

The IAM is inspired by self-attention mechanism [34,36], which estimates feature similarities for all pixels in a single image. While the self-attention mechanism finds self-similarity in a single image, the proposed IAM calculates the inter-similarity between LDR images for every pixel, which are used to align non-reference features toward the reference feature. In this section, we discuss the mechanism of the proposed IAM for the training and testing phase. Given two feature pairs $\left\{F_{r},F_{o}\right\}\in R^{C\times H\times W}$, they are reshaped as $R^{C\times HW}$. As shown in Figure 3, both pairs pass through the 1×1 convolutions for source $\theta_{s}$ and target $\theta_{t}$. By multiplying these two feature maps, a correlation map $C_{o\to r}\in R^{HW\times HW}$ is generated such that $C_{o\to r}=\theta_{t}{\left(F_{r}\right)}^{T}\theta_{s}\left(F_{o}\right)$. This correlation map is softmax normalized to generate a soft inter-attention map $A_{o\to r}\in R^{HW\times HW}$.

As the soft inter-attention map $A_{o\to r}$ is softmax normalized, it represents the matching probability for all spatial positions. However, in the optical flow algorithm, there is only one matching point for each pixel. To ensure that the inter-attention map represents only one matching point, a hard inter-attention map $B_{o\to r}\left(i,j\right)\in R^{HW\times HW}$ is generated as follows:

(1) $$B_{o\to r}\left(i,j\right)=\left\{\begin{array}{cc} 1, & for\;\forall i\;and\underset{{}^{j^{{}^{\prime }}}}{\arg\max}A_{o\to r}\left(i,j^{{}^{\prime }}\right)\\ 0, & otherwise. \end{array}\right.$$

With the hard inter-attention map $B_{o\to r}$, we can warp the other feature $F_{o}$ toward reference one $F_{r}$ using matrix multiplication, resulting in $F_{o}^{\prime }=B_{o\to r}F_{o}$. Finally, it is reshaped such that $F_{o}^{\prime }\in R^{C\times H\times W}$.

For training the IAM, we take the following additional steps. First, we generate an additional soft inter-attention map $A_{r\to o}$. We can train the IAM using photometric loss in an unsupervised manner, as described in Section 3.4. Photometric loss requires forward warping results using the soft inter-attention map. However, the occlusion problem, which originates from forward warping using an inter-attention map, is inevitable. An occluded region causes the network to estimate unreliable correspondences when using photometric loss for flow estimation [42] in an unsupervised manner.

To ensure that the alignment network estimates reliable correspondences, we generate a validation mask for training the network. As suggested in [38], pixels in occluded regions typically have small weights in the inter-attention map $A_{r\to o}$. We design the validation mask $V_{r\to o}\in R^{HW}$ for the reference image and it can be obtained as follows:

(2) $$V_{r\to o}\left(j\right)=\left\{\begin{array}{cc} 1, & if\;\sum_{i\in \left\{1,2,\ldots ,HW\right\}}A_{r\to o}\left(i,j\right)>\tau ,\\ 0, & otherwise, \end{array}\right.$$

where HW is a multiplication of the height and width of feature $F_{r}$ and τ is a threshold. Here, we set the τ to 0.1 empirically. In the same manner, the validation mask $V_{o\to r}$ can be generated. The validation masks $\left\{V_{r\to o},V_{o\to r}\right\}$ are used for photometric loss for training the IAM in an unsupervised manner, as described in Section 3.4.

#### 3.2.2. Pyramid Inter-Attention Module

**Figure 4:**
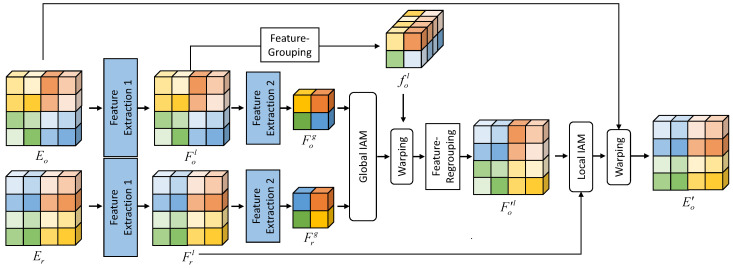
Illustration of pyramid inter-attention module (PIAM). The global IAM first finds global correspondences using discriminative down-sampled features $\left\{F_{r}^{g},F_{o}^{g}\right\}$ at a coarse level. After warping the first level feature $F_{o}^{l}$ toward the reference domain *r*, the local IAM finds local correspondences using features $\left\{F_{r}^{l},{F^{\prime }}_{o}^{l}\right\}$ at a fine level. Finally, we can warp the feature $E_{o}$ toward $E_{r}$ to generate ${E^{\prime }}_{o}$.

Finding global correspondences using the IAM for a large image requires a large amount of memory, which is described in Table 1. To alleviate this issue, we propose the PIAM, which consists of global IAM and local IAM, based on coarse-to-fine approaches for estimating correspondences [23,33]. As illustrated in Figure 4, feature pairs $\left\{E_{r},E_{o}\right\}\in R^{C\times H\times W}$ pass through two stages of feature extraction network. The first feature extraction network outputs feature pair $\left\{F_{r}^{l},F_{o}^{l}\right\}\in R^{C\times H\times W}$, the size of which is the same as the resolution of $\left\{E_{r},E_{o}\right\}$. The second network, which consists of *n* convolutions with stride-2, outputs feature pair $\left\{F_{r}^{g},F_{o}^{g}\right\}\in R^{C\times \left(H/2^{n}\right)\times \left(W/2^{n}\right)}$.

The global-IAM first estimates $B_{o\to r}^{g}\in R^{\left(HW/2^{2n}\right)\times \left(HW/2^{2n}\right)}$, which represents global correspondences, using the down-sampled features $\left\{F_{r}^{g},F_{o}^{g}\right\}$. While other deep learning methods using coarse-to-fine approaches warp features $\left\{F_{r}^{l},F_{o}^{l}\right\}$ using up-sampled correspondences, we directly use global the correspondences $B_{o\to r}^{g}$. To match the size, we generate $f_{o}^{l}\in R^{C\cdot 2^{2n}\times \left(H/2^{n}\right)\times \left(W/2^{n}\right)}$ by performing feature-grouping on the features $F_{o}^{l}\in R^{C\times H\times W}$ as shown in Figure 4. The feature-grouping operation first divides feature $F_{o}^{l}\in R^{C\times H\times W}$ into grid of patches whose shape is $R^{C\times 2^{n}\times 2^{n}}$ and reshape each patch to the size of $R^{C\cdot 2^{2n}\times 1\times 1}$, then combines these patches to make $f_{o}^{l}\in R^{C\cdot 2^{2n}\times \left(H/2^{n}\right)\times \left(W/2^{n}\right)}$. The coarse-globally aligned feature ${F^{\prime }}_{o}^{l}$ is generated by performing feature-regrouping, which is the inverse operation of feature-grouping, on warped first level feature $B_{o\to r}^{g}f_{o}^{l}$.

Finally, we can find the local correspondence between the feature pair $\left\{F_{r}^{l},{F^{\prime }}_{o}^{l}\right\}$. To reduce the computational memory, in the local IAM, we divide both features $\left\{F_{r}^{l},{F^{\prime }}_{o}^{l}\right\}$ into grids of patches such that the size of the patches is k×k, and then perform alignment with local patches to find local correspondences. We divide a feature into a grid, such that $F_{r}^{l,n}=\left\{F_{i}^{l,1},\ldots ,F_{i}^{l,N}\right\}$, where N=(H/k)·(W/k) is the number of patches. It should be noted that $F^{l,n}$ denotes the *n*-th patch consisting of $F^{l}$. The local IAM takes each input pairs $\left\{F_{r}^{l,n},{F^{\prime }}_{o}^{l,n}\right\}$, and outputs local correspondence $B_{o\to r}^{l,n}$. With these local correspondences, we finally generate warped feature $E_{o}^{\prime }$.

### 3.3. Merging Network

**Figure 5:**
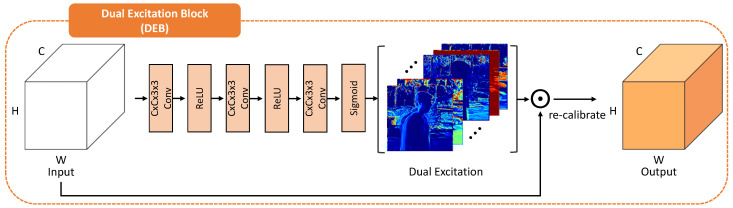
Illustration of dual excitation block (DEB), which outputs dual excitations. Fused features are recalibrated both spatially and channel-wise to highlight the meaningful features and exclude harmful features caused by alignment error or saturation.

After aligning other features $\left\{E_{1},E_{3}\right\}$ to the reference feature $E_{2}$ using the alignment network, we obtain the warped features $\left\{E_{1}^{\prime },E_{3}^{\prime }\right\}$. Despite the alignment process based on PIAM, the alignment error that PIAM cannot handle still exists. In order to eliminate the harmful effect of features in a region of misalignment or saturation, we designed a novel network that incorporates the dual excitation block (DEB) (Figure 5) and dilated residual dense block (DRDB) [21] during the merging process. Finally, the ghost-free HDR images are generated by reducing artifacts caused by misalignment and preserving details during the merging process.

#### 3.3.1. Dual Excitation Block (DEB)

In contrast to other non-flow-based deep HDR methods [20,21], which only fuses misaligned features $E_{1}$, $E_{2}$, $E_{3}$, we fuse warped features using the PIAM. As shown in Figure 5, the input of the DEB is a fusion of warped features and a reference feature. Feature fusion is defined as follows:

(3) $$G_{fuse}=\mathrm{Concat}\left(E_{1}^{\prime },E_{2},E_{3}^{\prime }\right)$$

where Concat() is a concatenation operation.

The DEB recalibrates the fused feature $G_{fuse}\in R^{C\times H\times W}$ both spatially and channel-wise by multiplying its excitation. Excitation allocates weights spatially and channel-wise to the fused feature to suppress the harmful features and encourage informative features for generating ghost-free HDR images. The configuration of the DEB is illustrated in Figure 5. After $G_{fuse}$ passes several convolutions followed by ReLU functions and a sigmoid function, the DEB finally generates dual excitations. We can recalibrate fused feature by multiplying the excitation. Unlike the attention of Yan [21], we calculate both spatial and channel-wise excitation to refine fused features, whereas attention only represents the spatial excitation that the DEB outputs.

#### 3.3.2. Dilated Residual Dense Block (DRDB)

The DRDB consists of dilated convolutions to facilitate large receptive field for acquiring additional contextual information. The residual and dense connections in the DRDB enable us to use all of the hierarchical features contained in fused features. The details of the DRDB are described in [21].

### 3.4. Training Losses

The proposed method consists of two tasks: alignment and HDR generation. We designed a loss function for training the alignment task that finds the correspondences between LDR images. Based on the procedure described in [19,20,21], we also use the HDR reconstruction loss. The overall loss function is defined as follows:

(4) $$L=\lambda L^{align}+L^{HDR},$$

where λ controls the ratio of training alignment among the overall loss. λ was empirically set to 0.5.

#### 3.4.1. Alignment Loss

Since there are no labeled dense correspondences between LDR images in an HDR dataset, we train the PIAM in an unsupervised manner. We introduce photometric loss for training the alignment network, following [38,43]. Photometric loss works for the images with the same exposure value. However, in our case, the LDR images have different exposures. Therefore, we set the same brightness values, as suggested in [19]. The brightness constancy is maintained by raising the exposure of darker images to that of brighter images. For example, if $I_{1}$ is darker than $I_{2}$, then their exposures are matched such that $M_{1}=\mathrm{clip}\left(I_{1}{\left(t_{2}/t_{1}\right)}^{1/\gamma }\right)$ and $M_{2}=I_{2}$, where clip ensures the range of the output is $\left[0,1\right]$, $t_{1}$ and $t_{2}$ are the exposure times of $I_{1}$ and $I_{2}$, respectively.

With exposure-corrected matched pairs $\left\{M_{s},M_{t}\right\}$, the PIAM can be trained using the soft inter-attention maps $A_{s\to t}$ in an unsupervised manner by minimizing photometric error in valid region $V_{s\to t}$. To train the global IAM using $\left\{M_{s},M_{t}\right\}$, we define global alignment loss such that:

(5) $$L_{s\to t}^{global}=\frac{\sum_{p}{∥\left(A_{s\to t}^{g}m_{s}\left(p\right)-m_{t}\left(p\right)\right)⊙V_{s\to t}^{g}\left(p\right)∥}_{1}}{\sum_{p}{∥V_{s\to t}^{g}\left(p\right)∥}_{1}},$$

where *s* denotes a source, *t* denotes a target, ⊙ denotes element-wise multiplication and *m* is generated by feature-grouping on *M*. The global IAM first warps $M_{s}$ to $M_{t}$ globally, generating $M_{s}^{\prime }$. We can train the local IAM using the local alignment loss as follows:

(6) $$L_{s\to t}^{local}=\sum_{n}\left(\frac{\sum_{p}{∥\left(A_{s\to t}^{l,n}{M^{\prime }}_{s}^{n}\left(p\right)-M_{t}^{n}\left(p\right)\right)⊙V_{s\to t}^{l,n}\left(p\right)∥}_{1}}{\sum_{p}{∥V_{s\to t}^{l,n}\left(p\right)∥}_{1}}\right),$$

where *s* denotes a source, *t* denotes a target, and ⊙ denotes element-wise multiplication. In this work, we set the reference *r* to 2, and other *o* to 1 or 3. Therefore, the overall alignment loss for training the PIAM is defined as follows.

(7) $$L^{align}=L_{1\to 2}^{global}+L_{2\to 1}^{global}+L_{3\to 2}^{global}+L_{2\to 3}^{global}+L_{1\to 2}^{local}+L_{2\to 1}^{local}+L_{3\to 2}^{local}+L_{2\to 3}^{local}.$$

#### 3.4.2. HDR Reconstruction Loss

Since the HDR images are usually displayed after tonemapping, the proposed HDR imaging network estimates a tonemapped HDR image *H* using the μ-law described in [19] as follows:

(8) $$T\left(H\right)=\frac{\log\left(1+\mu H\right)}{\log\left(1+\mu \right)},$$

where μ is a parameter that controls the amount of compression. In this work, we set μ to 5000. This tonemapping function is differentiable, which facilitates the training of our model in an end-to-end manner. The loss function for estimating an HDR image *H* with $H^{gt}$ is defined as follows:

(9) $$L^{HDR}={∥T\left(H\right)-T\left(H^{gt}\right)∥}_{1}.$$

## 4. Experiments

### 4.1. Implementation Details

All convolutional filters in feature extraction network are 3×3 filters, followed by ReLU functions. In the PIAM, the second level feature extraction network consists of three convolutions for 8× down-sampling. For local the IAM, we set the size of the local patch to 32×32 for both training and testing. The growth rate was set to 32 in the DRDB. Our network was implemented using Pytorch on a PC with an Nvidia RTX 2080 GPU. The network was trained using the Adam optimizer [44] with $\beta_{1}=0.9$, $\beta_{2}=0.99$. The HDR imaging network was trained with a batch size of one and learning rate $1\times 10^{-5}$, respectively. Data augmentation was performed by flipping the images or performing color channel swapping in the images. During training, the input images were randomly cropped to a size of 256×256 pixels. Training was completed after 200,000 iterations, when additional iterations could not provide any further improvements for alignment or HDR imaging. All methods including our method were implemented to produce 640×960 HDR images in the experiments.

### 4.2. Experimental Settings

#### 4.2.1. Datasets

The proposed HDR imaging network was trained using Kalantari’s HDR dataset [19] according to the process presented in previous works [19,20,21]. Kalantari’s HDR dataset provides ground truth HDR images, which facilitate training an HDR imaging network in a supervised manner. It consists of 74 sets for training and 15 sets for testing. Each set consists of three LDR images captured with different exposure values ($\left\{-2,0+2\right\}$ or $\left\{-3,0+3\right\}$) and the ground truth HDR image is aligned to the reference image (middle exposure). The details of constructing the ground truth HDR image are discussed in [19]. After training our network on Kalantari’s HDR dataset [19], we compared the performance of our HDR imaging method with that of other state-of-the-art methods by testing on this dataset both qualitatively and quantitatively. We also used Sen’s dataset [11] and Tursun’s dataset [24] for visual comparisons since they do not contain ground truth HDR images.

#### 4.2.2. Evaluation Metrics

We compared our method with the various state-of-the-art methods quantitatively on Kalantari’s dataset [19] because ground truth HDR images are available for this dataset. The evaluation metrics selected for measuring the quality of HDR images were PSNR-μ. PSNR-M, PSNR-L, and HDR-VDP-2. We computed the PSNR-μ values between the generated HDR images and ground truth HDR images after tonemapping using μ law. Additionally, evaluation metrics based on Matlab’s tonemap function (PSNR-M) and linear domains (PSNR-L) were adopted. To focus on the visual quality of HDR iamges, we also measured HDR-VDP-2 values [45].

### 4.3. Comparison with the State-of-the-Art Methods

We compare our method with the recent state-of-the-art methods, including hand-crafted [11,15,28] and CNN-based methods [17,19,20,21,22], on Kalantari et al.’s dataset [19] in Section 4.4 and datasets without ground truth images [11,24] in Section 4.5. For fair comparison, we used the same environment such as training dataset and implementation details for CNN-based methods [17,19,20,21,22]. All results were obtained using the code provided by the original authors.

### 4.4. Experiments on Kalantari et al.’s Dataset

#### 4.4.1. Qualitative Comparison

**Figure 6:**
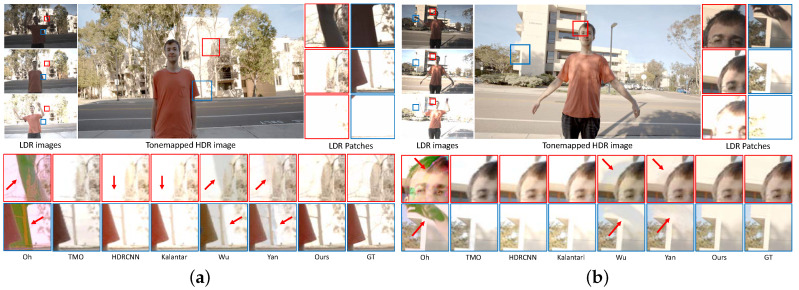
Visual comparisons on (**a**) testing 007 data and (**b**) testing 008 data from from Kalantari’s dataset [19]. In the top section, we present the input LDR images, tonemapped HDR image produced by the proposed method, and LDR image patches. In the bottom section, we compare magnified local patches of the HDR images generated by our method and the state-of-the-art methods. Our network produces high-quality HDR images in the presence of saturation and object motions.

Figure 6 presents visual comparisons of HDR images for the proposed method and the state-of-the-art methods on the testing set of the Kalantari HDR dataset [19]. The method proposed by

Oh et al. [28] cannot detect object motion, resulting in large ghosting artifacts due to the misalignment. Especially, the results of Oh et al. [28] are strongly influenced by LDR images with low exposure values. HDR imaging methods using single images, such as TMO [17] and HDRCNN [22], cannot elucidate the details of ground truth HDR images, since they only use a single reference image. Among the CNN-based methods for fusing LDR images, Wu et al. [20] and Yan et al. [21] do not conduct alignment prior to merging. Therefore, they suffer from ghosting artifacts caused by misalignment. The method proposed by Yan et al. [21] generates more plausible results than that proposed by Wu et al. because it uses attention maps, which is a similar mechanism to our spatial excitations. Although the method proposed by Kalantari et al. [19] conducts alignment prior to merging, it produces saturated results because it cannot suppress harmful features during the merging process. In contrast, our method is free from any artifacts, resulting in more plausible results than any other method, since we conduct alignment and recalibrate features by levering the PIAM and DEB.

#### 4.4.2. Quantitative Comparison

We measured the performance of recent state-of-the-art methods and our method for quantitative evaluation on Kalantari HDR dataset [19]. We tested 15 images from testing dataset, measured the all evaluation metrics described above, and calculated average values. The results are presented in Table 2. In terms of all of the evaluation metrics, our method yields the best HDR imaging results. This is mainly because our method can fully exploit the all LDR features through alignment and recalibrate features for highlighting the informative features and excluding harmful components.

### 4.5. Experiments on Datasets without Ground Truth

#### Qualitative Comparison

**Figure 7:**
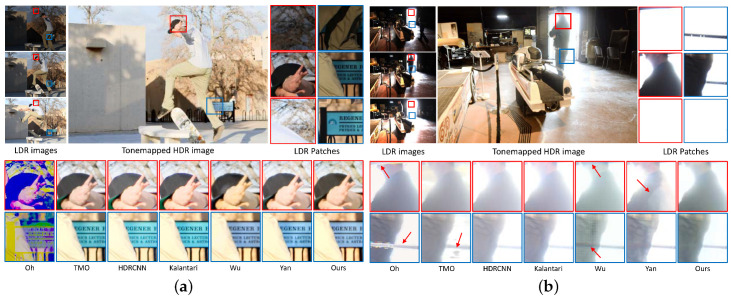
Visual comparisons on (**a**) Sen et al.’s dataset Testing data (skater) from [11] and (**b**) Tursun et al.’s dataset Testing data (53) from [24]. In the top section, we present the input LDR images, tonemapped HDR images produced by the proposed method, and LDR image patches. In the bottom section, we compare magnified patches of the HDR images generated by our method and the state-of-the-art methods. Ground truths are not included because these datasets do not provide them. The proposed method yields plausible results without ghosting artifacts or color distortions.

Figure 7 presents visual comparisons of HDR images for the proposed method and the state-of-the-art methods on the testing set of datasets without ground truth [11,24]. Oh et al.’s [28] method cannot detect large object motion, resulting in large ghosting artifacts in Figure 7a. The methods relying on single images [17,22] and Kalantari et al.’s method [19] exhibit similar color distortions for both datasets. Wu et al.’s method [20] yields color distortions and ghosting artifacts. The method proposed by Yan et al. [21] fails to preserve color consistency and generates ghost artifacts due to misalignment. In contrast, our method generates visually plausible results preserves details and color consistency without ghosting artifacts.

### 4.6. Analysis

#### 4.6.1. Ablation Studies

**Figure 8:**
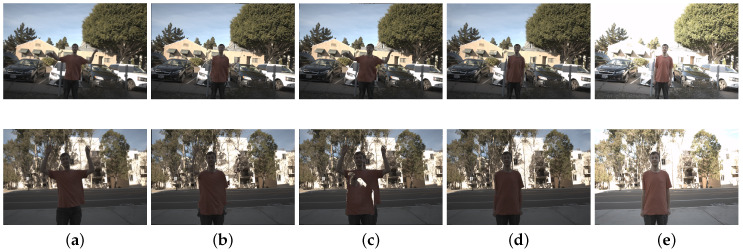
Visual comparisons of alignment methods. We conducted alignment using various alignment algorithms for warping (**a**) Non-reference LDR towards (**e**) Reference LDR $I_{2}$. The warping results are presented in (**b**) SIFTflow [23], (**c**) PWC-Net [33] © IEEE 2020, (**d**) our method.

To verify the effectiveness of our network architecture, we conducted ablation studies to quantify the effects of the proposed pyramid inter-attention module (PIAM) and dual excitation block (DEB). Table 3 compares the performances of HDR imaging networks with different components in terms of the target evaluation metrics. It can be observed that all of the evaluation metrics decrease where the PIAM or DEB are not applied in our network. (i.e., baseline network). As shown in Figure 8, the PIAM finds reliable correspondences between LDR features. By conducting alignment using the PIAM, performance increases because the PIAM enables the network to exploit well-aligned LDR features by providing more precise information to the merging network. Furthermore, the DEB also increases the performance of HDR imaging because it can re-calibrate features both spatially and channel-wise to boost the representation power of fused features for reconstructing a HDR image. Therefore, it refines fused features to make them more informative, resulting in improved performance. With the PIAM and DEB added to the baseline network, our method achieves the best performance. The PIAM boosts the performance by providing more precise information and the DEB boosts performance by recalibrating features.

#### 4.6.2. Matching Accuracy Comparison

To demonstrate the superiority of our alignment process using the PIAM for HDR imaging, we compared our method with the conventional optical flow algorithm [23] and the deep-learning-based flow estimation method [33], by measuring the accuracy of these correspondence methods. To measure matching accuracy, we compare the structural difference between warped images and reference LDR images on testing set in Kalantari et al.’s dataset. Since the intensity of a reference-warped LDR image is different from that of the LDR reference image, we compared SSIM values. Figure 8 presents a qualitative comparison of alignment results for our method, SIFT-flow [23], and PWC-Net [33]. As shown in Figure 8, PWC-Net fails to find large correspondences between LDR images because it is designed to cover small displacement. Although SIFT-flow finds large correspondences, it cannot preserve the details around the boundary of the moving object in the warped image. In contrast to these methods, our method yields more reliable correspondences. In Table 4, it can be seen that the proposed PIAM yields more accurate alignment performance than conventional the optical flow algorithm [23] used in Kalantari et al.’s [19], resulting in enhanced performance for HDR imaging.

#### 4.6.3. Run Time Comparison

Table 5 presents the run time comparisons between various methods. All algorithms were executed on a PC with an i7-4790K (4.0GHz) CPU, 28 GB of RAM, and an Nvidia RTX 2080 GPU. It should be noted that the optimization-based HDR method [28] and HDR method [19] using the classical optical flow algorithm [23] were executed using the CPU. Our method is slower than the other deep-learning-based method except for Kalantari et al.’s method, which uses the conventional optical flow algorithm. Although the PIAM in our method increases the run time, it is still approximately 60 times faster than Kalantari et al.’s method. It should be noted that the other methods that are faster than our method do not contain alignment processes, resulting in the ghosting artifacts. Even though we conduct an alignment process similar to Kalantari et al.’s process, our method finds correspondences between LDR images more efficiently and effectively.

#### 4.6.4. Cellphone Example

**Figure 9:**
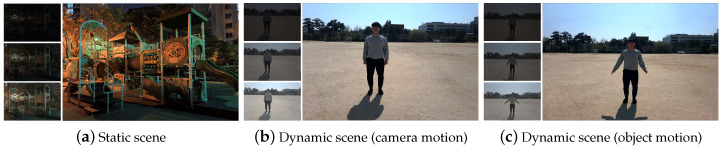
HDR results of using cellphones to capture for both static (camera, object motion) and dynamic scenes. HDR results are aligned to the middle exposure. All LDR images were captured using a Samsung Galaxy S20 device.

We also tested our method on cellphone images of both static and dynamic scenes to verify its practicality. For dynamic scenes, we tested with different types of motions such as camera motion or object motion. The HDR results are presented in Figure 9. One can see that our network produces plausible results in various types of settings. The LDR images were captured using a Samsung Galaxy S20 device with different exposure values. The exposure values for the cellphone were $\left\{-4,-2,0\right\}$, which are different from training settings for the proposed method. Even with different settings, the plausible results demonstrate the robustness of our network.

## 5. Conclusions

We developed a novel end-to-end approach to reconstructing ghost-free HDR images of dynamic scenes. The proposed PIAM effectively aligns LDR features to exploit all LDR features for HDR reconstruction, even when large motion exists. Additionally, the DEB recalibrates the aligned features by multiplying the excitations spatially and channel-wise to boost the representation power of them. Ablation studies clearly demonstrated the effectiveness of the PIAM and DEB of our model. Finally, we have demonstrated that the proposed method is robust to dynamic scenes with large foreground motion, and outperforms state-of-the-art methods on standard benchmarks by a significant margin.

## Figures and Tables

**Table 1:** Total memories of soft inter-attention map to find global correspondence.

	Size of Tensors	H=256,W=256,n=3,k=32	H=640,W=960,n=3,k=32
IAM	(HW)×(HW)	4,294,967,296	377,487,360,000
PIAM	$\left(HW/2^{2n}\right)\times \left(HW/2^{2n}\right)+\left(H/k\right)\times \left(W/k\right)\times \left(k\right)\times \left(k\right)$	16 + 65,536	9,600 + 614,400

**Table 2:** Quantitative comparisons of the proposed method with state-of-the-art methods on [19], where bold indicates the best performance.

	PSNR-μ	PSNR-M	PSNR-L	HDR-VDP-2
Sen [11]	40.924	30.572	37.934	55.145
Hu [15]	32.021	24.982	30.610	55.104
Oh [28]	26.151	21.051	25.131	45.526
TMO [17]	8.612	24.384	7.904	43.394
HDRCNN [22]	14.387	24.503	13.704	46.790
Kalantari [19]	41.170	30.705	40.226	59.909
Wu [20]	39.345	31.159	38.782	59.296
Yan [21]	42.017	31.798	40.978	61.104
Ours	43.212	32.415	41.697	62.481

**Table 3:** Ablation study on different components of the proposed HDR imaging network.

		PSNR-μ	PSNR-M	PSNR-L	HDR-VDP-2
	baseline network	38.514	31.475	38.021	58.457
	+PIAM	41.824	31.595	39.945	60.184
	+DEB	41.524	31.518	40.211	60.858
	+PIAM +DEB	43.212	32.415	41.697	62.481

**Table 4:** Quantitative evaluation for matching accuracy in [19].

	W/o Alignment	SIFT-Flow [23]	PWC-Net [33]	PIAM
SSIM	0.4326	0.6342	0.6042	0.6614

**Table 5:** Run time (in seconds) for different methods averaged on test images [19], where ‘*’ denotes the methods using CPU. Other methods are tested under GPU environment.

	Oh * [28]	HDRCNN [22]	Kalantari * [19]	Wu [20]	Yan [21]	Ours
Times (s)	65.153	0.245	40.518	0.215	0.301	0.594

## References

[1] Banterle F., Artusi A., Debattista K., Chalmers A. (2017). Advanced High Dynamic Range Imaging.

[2] Mann S., Rosalind W. On being undigital with digital cameras: Extending dynamic range by combining exposed pictures. Proceedings of the IST 48th Annual Conference Society for Imaging Science and Technology.

[3] Debevec P.E., Malik J. (2008). Recovering high dynamic range radiance maps from photographs. ACM SIGGRAPH.

[4] Zhang W., Cham W.K. (2011). Gradient-directed multiexposure composition. IEEE Trans. Image Process..

[5] Jacobs K., Loscos C., Ward G. (2008). Automatic high-dynamic range image generation for dynamic scenes. IEEE Comput. Graph. Appl..

[6] Grosch T. (2006). Fast and robust high dynamic range image generation with camera and object movement. Vision, Model. Vis. Rwth Aachen.

[7] Pece F., Kautz J. Bitmap movement detection: HDR for dynamic scenes. Proceedings of the 2010 Conference on Visual Media Production.

[8] Heo Y.S., Lee K.M., Lee S.U., Moon Y., Cha J. Ghost-free high dynamic range imaging. Proceedings of the Asian Conference on Computer Vision.

[9] Kang S.B., Uyttendaele M., Winder S., Szeliski R. (2003). High dynamic range video. ACM Trans. Graph. (TOG).

[10] Bogoni L. Extending dynamic range of monochrome and color images through fusion. Proceedings of the 15th International Conference on Pattern Recognition (ICPR-2000).

[11] Sen P., Kalantari N.K., Yaesoubi M., Darabi S., Goldman D.B., Shechtman E. (2012). Robust patch-based hdr reconstruction of dynamic scenes. ACM Trans. Graph. (TOG).

[12] Hafner D., Demetz O., Weickert J. Simultaneous HDR and optic flow computation. Proceedings of the 2014 22nd International Conference on Pattern Recognition.

[13] Tomaszewska A., Mantiuk R. Image registration for multi-exposure high dynamic range image acquisition. Proceedings of the 15th International Conference in Central Europe on Computer Graphics, Visualization and Computer Vision 2007 in co-operation with EUROGRAPHICS.

[14] Gallo O., Troccoli A., Hu J., Pulli K., Kautz J. Locally non-rigid registration for mobile HDR photography. Proceedings of the IEEE Conference on Computer Vision and Pattern Recognition Workshops.

[15] Hu J., Gallo O., Pulli K., Sun X. HDR deghosting: How to deal with saturation?. Proceedings of the IEEE Conference on Computer Vision and Pattern Recognition.

[16] Zimmer H., Bruhn A., Weickert J. (2011). Freehand HDR imaging of moving scenes with simultaneous resolution enhancement. Comput. Graph. Forum.

[17] Endo Y., Kanamori Y., Mitani J. (2017). Deep reverse tone mapping. ACM Trans. Graph. (TOG).

[18] Jung H., Kim Y., Jang H., Ha N., Sohn K. (2020). Unsupervised Deep Image Fusion With Structure Tensor Representations. IEEE Trans. Image Process..

[19] Kalantari N.K., Ramamoorthi R. (2017). Deep high dynamic range imaging of dynamic scenes. ACM Trans. Graph. (TOG).

[20] Wu S., Xu J., Tai Y.W., Tang C.K. Deep high dynamic range imaging with large foreground motions. Proceedings of the European Conference on Computer Vision.

[21] Yan Q., Gong D., Shi Q., Hengel A.V.D., Shen C., Reid I., Zhang Y. Attention-guided Network for Ghost-free High Dynamic Range Imaging. Proceedings of the IEEE Conference on Computer Vision and Pattern Recognition.

[22] Eilertsen G., Kronander J., Denes G., Mantiuk R.K., Unger J. (2017). HDR image reconstruction from a single exposure using deep CNNs. ACM Trans. Graph. (TOG).

[23] Liu C. (2009). Beyond Pixels: Exploring New Representations and Applications for Motion Analysis. Ph.D. Thesis.

[24] Tursun O.T., Akyüz A.O., Erdem A., Erdem E. (2016). An objective deghosting quality metric for HDR images. Comput. Graph. Forum.

[25] Unger J., Gustavson S. (2007). High-dynamic-range video for photometric measurement of illumination. Sensors, Cameras, and Systems for Scientific/Industrial Applications VIII.

[26] Khan E.A., Akyuz A.O., Reinhard E. Ghost removal in high dynamic range images. Proceedings of the 2006 International Conference on Image Processing.

[27] Lee C., Li Y., Monga V. (2014). Ghost-free high dynamic range imaging via rank minimization. IEEE Signal Process. Lett..

[28] Oh T.H., Lee J.Y., Tai Y.W., Kweon I.S. (2014). Robust high dynamic range imaging by rank minimization. IEEE Trans. Pattern Anal. Mach. Intell..

[29] Jinno T., Okuda M. Motion blur free HDR image acquisition using multiple exposures. Proceedings of the 2008 15th IEEE International Conference on Image Processing.

[30] Dosovitskiy A., Fischer P., Ilg E., Hausser P., Hazirbas C., Golkov V., Brox T. Flownet: Learning optical flow with convolutional networks. Proceedings of the IEEE International Conference on Computer Vision.

[31] Ronneberger O., Fischer P., Brox T. U-net: Convolutional networks for biomedical image segmentation. Proceedings of the International Conference on Medical Image Computing and Computer-Assisted Intervention.

[32] Ilg E., Mayer N., Saikia T., Keuper M., Dosovitskiy A., Smagt P.V.D., Cremers D., Brox T. Flownet 2.0: Evolution of optical flow estimation with deep networks. Proceedings of the IEEE Conference on Computer Vision and Pattern Recognition.

[33] Sun D., Yang X., Liu M.Y., Kautz J. Pwc-net: Cnns for optical flow using pyramid, warping, and cost volume. Proceedings of the IEEE conference on computer vision and pattern recognition.

[34] Wang X., Girshick R., Gupta A., He K. Non-local neural networks. Proceedings of the IEEE conference on computer vision and pattern recognition.

[35] Fu J., Liu J., Tian H., Li Y., Bao Y., Fang Z., Lu H. Dual attention network for scene segmentation. Proceedings of the IEEE Conference on Computer Vision and Pattern Recognition.

[36] Zhang H., Goodfellow I., Metaxas D., Odena A. Self-attention generative adversarial networks. Proceedings of the International Conference on Machine Learning.

[37] Li G., He X., Zhang W., Chang H., Dong L., Lin L. Non-locally enhanced encoder-decoder network for single image de-raining. Proceedings of the 26th ACM International Conference on Multimedia.

[38] Wang L., Wang Y., Liang Z., Lin Z., Yang J., An W., Guo Y. Learning parallax attention for stereo image super-resolution. Proceedings of the IEEE Conference on Computer Vision and Pattern Recognition.

[39] Chang J.R., Chen Y.S. Pyramid stereo matching network. Proceedings of the IEEE Conference on Computer Vision and Pattern Recognition.

[40] Kendall A., Martirosyan H., Dasgupta S., Henry P., Kennedy R., Bachrach A., Bry A. End-to-end learning of geometry and context for deep stereo regression. Proceedings of the IEEE International Conference on Computer Vision.

[41] Liang Z., Feng Y., Guo Y., Liu H., Chen W., Qiao L., Zhang J. Learning for disparity estimation through feature constancy. Proceedings of the IEEE Conference on Computer Vision and Pattern Recognition.

[42] Wang Y., Yang Y., Yang Z., Zhao L., Wang P., Xu W. Occlusion aware unsupervised learning of optical flow. Proceedings of the IEEE Conference on Computer Vision and Pattern Recognition.

[43] Godard C., Mac Aodha O., Brostow G.J. Unsupervised monocular depth estimation with left-right consistency. Proceedings of the IEEE Conference on Computer Vision and Pattern Recognition.

[44] Kingma D.P., Ba J. Adam: A method for stochastic optimization. Proceedings of the 3rd International Conference for Learning Representations.

[45] Mantiuk R., Kim K.J., Rempel A.G., Heidrich W. (2011). HDR-VDP-2: A calibrated visual metric for visibility and quality predictions in all luminance conditions. ACM Trans. Graph. (TOG).

